# Supplementary material for: Electrical Stimulation Generates Induced Tumor-Suppressing Cells, Offering a Potential Option for Combatting Breast Cancer and Bone Metastasis
Source: Int J Mol Sci. 2025 Jan 25;26(3):1030. doi: 10.3390/ijms26031030 (PMC11817334; doi:10.3390/ijms26031030)
Supplement: Supplementary file 1 [file ijms-26-01030-s001.zip › ijms-3384541-supplementary.pdf]

Supplementary Figures

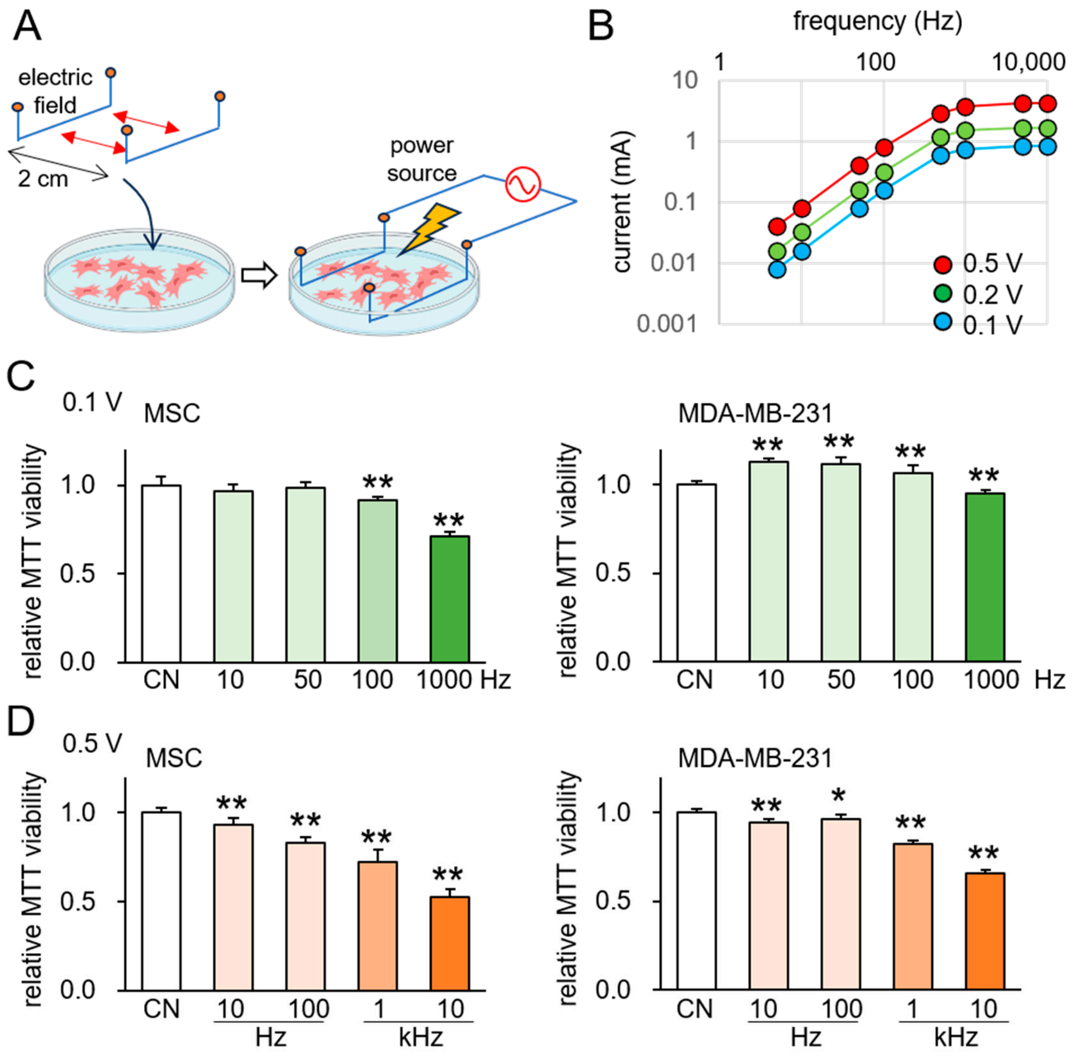

Figure S1. Effect of electrical stimulation (ES) on MSCs and MDA-MB-231 breast cancer cells. CN = control. Data are presented as mean ± SD (n = 6 independent experiments). Statistical analysis was performed using one-way ANOVA followed by Bonferroni correction. The asterisks indicate statistical significance compared to the control group. Single asterisk (*) indicates $p<0.05$, and double asterisks (**) indicate $p<0.01$. (A) Experimental setup with a pair of platinum wires and a power source. (B) Relationship between the frequency (Hz) and current (mA) under the source voltage at 0.1, 0.2, and 0.5 V. (C) MTT-based viability of MSCs and MDA-MB-231 cells in response to 0.1 V ES at 10 Hz to 1 kHz. (D) MTT-based viability of MSCs and MDA-MB-231 cells in response to 0.5 V ES at 10 Hz to 10 kHz.

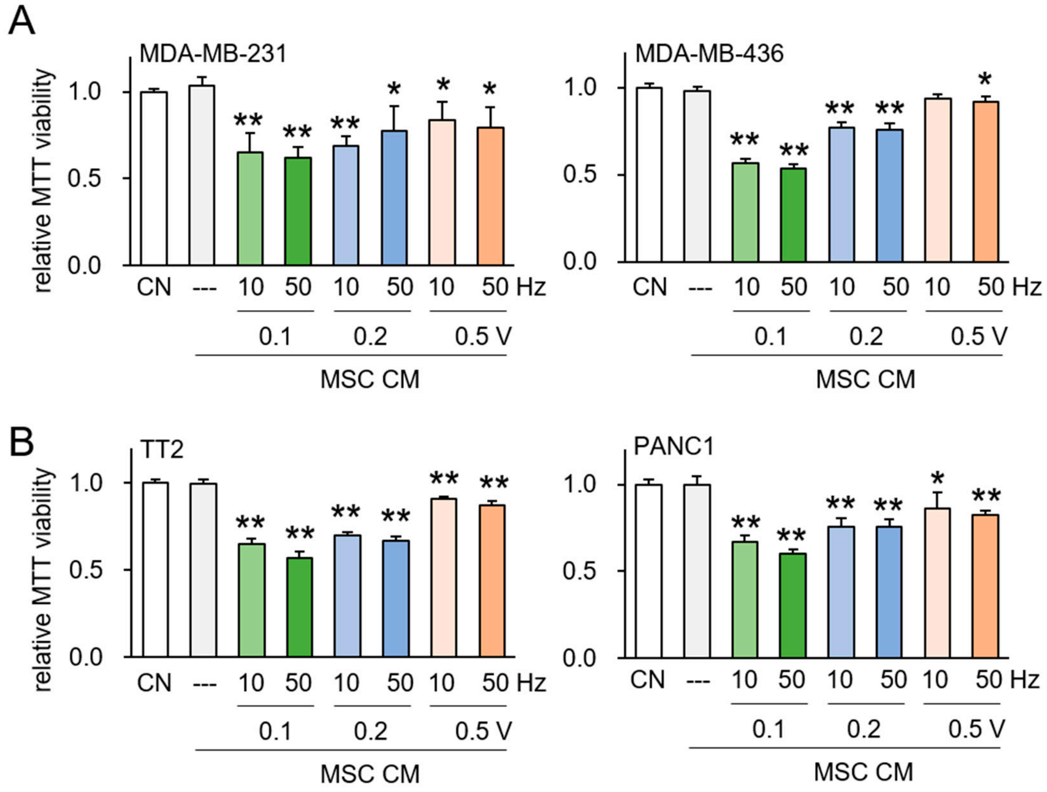

Figure S2. Tumor-suppressive effects of ES-treated MSCs. CN = control, and CM = conditioned medium. Data are presented as mean ± SD (n = 6 independent experiments). Statistical analysis was performed using one-way ANOVA followed by Bonferroni correction. The asterisks indicate statistical significance compared to the control group. Single asterisk (*) indicates $p<0.05$, and double asterisks (**) indicate $p<0.01$. ES was applied with 0.1, 0.2, and 0.5 V at 10 and 50 Hz for 1 h, and the CM was harvested 24 h later. (A) MTT-based viability of MDA-MB-231 and MDA-MB-436 breast cancer cells in response to ES-treated MSC CM. (B) MTT-based viability of TT2 OS and PANC1 PDAC cells in response to ES-treated MSC CM.

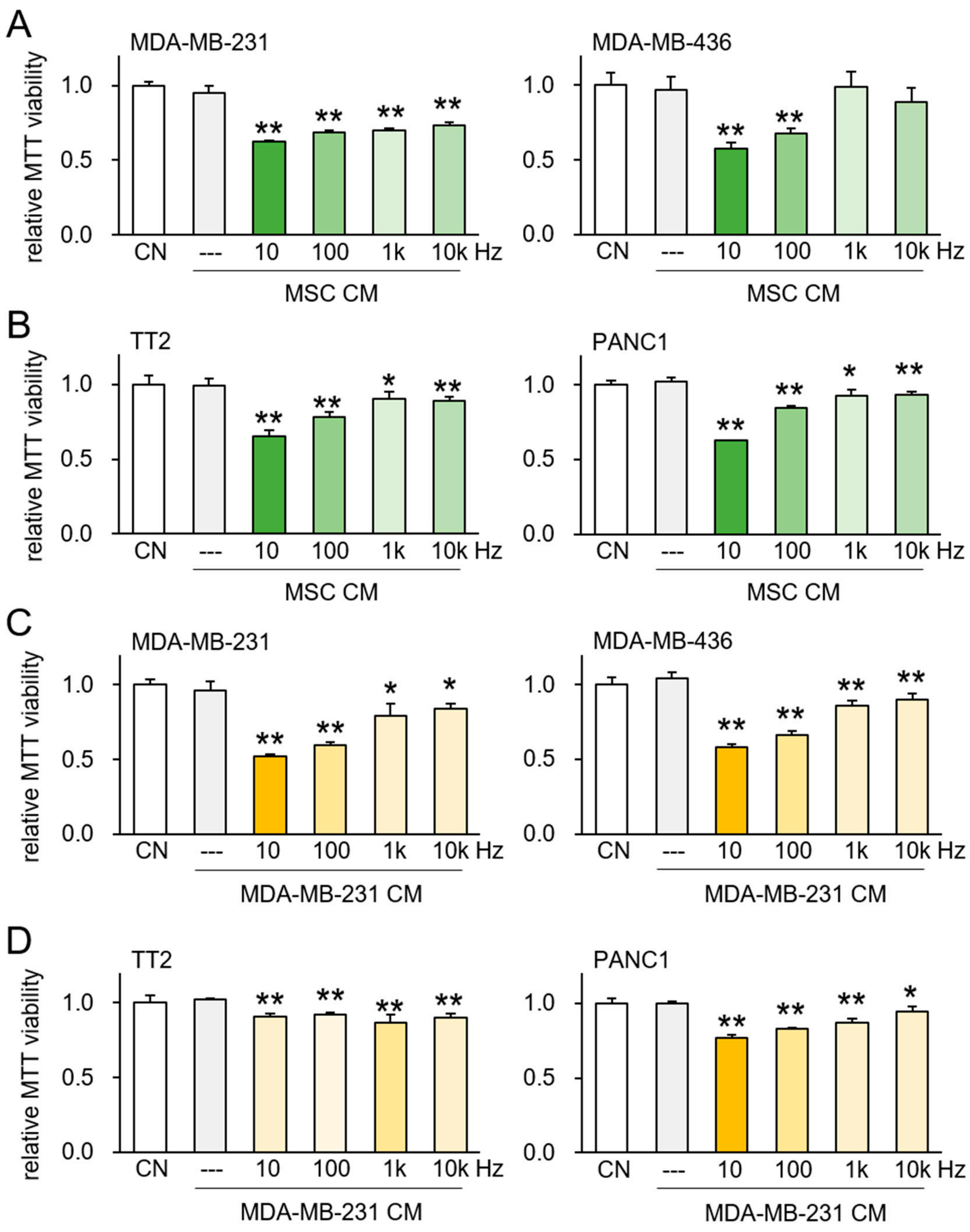

Figure S3. Tumor-suppressive effects of ES-treated MSC CM and MDA-MB-231 CM. CN = control, and CM = conditioned medium. Data are presented as mean ± SD (n = 6 independent experiments). Statistical analysis was performed using one-way ANOVA followed by Bonferroni correction. The asterisks indicate statistical significance compared to the control group. Single asterisk (*) indicates $p<0.05$, and double asterisks (**) indicate $p<0.01$. ES was applied with 0.1 V at 10 Hz to 10 kHz for 1 h, and the CM was harvested 24 h later. (A&B) MTT-based viability of 4 cancer cell lines (MDA-MB-231, MDA-MB-436, TT2, and PANC1) in response to ES-treated MSC CM. (C&D) MTT-based viability of 4 cancer cell lines (MDA-MB-231, MDA-MB-436, TT2, and PANC1) in response to ES-treated MDA-MB-231 CM.

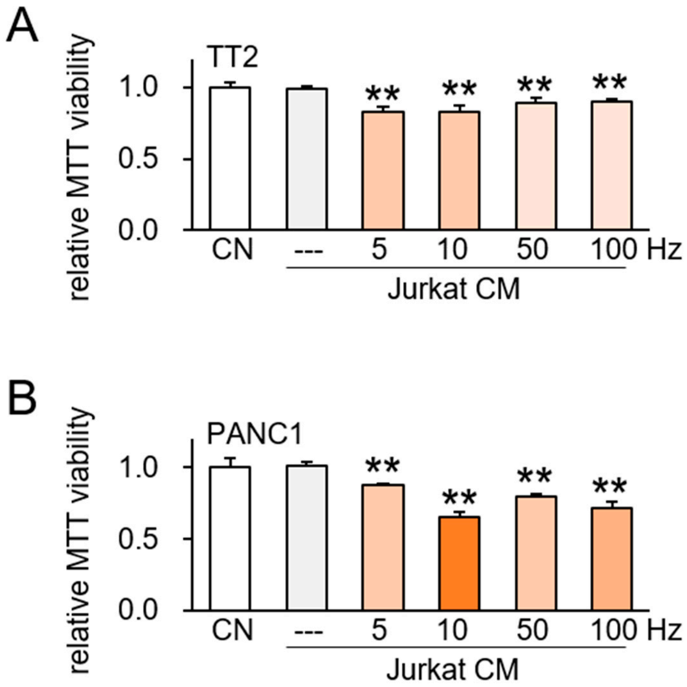

Figure S4. Tumor-suppressive effects of ES-treated Jurkat CM. CN = control, and CM = conditioned medium. Data are presented as mean ± SD (n = 6 independent experiments). Statistical analysis was performed using one-way ANOVA followed by Bonferroni correction. The asterisks indicate statistical significance compared to the control group. Single asterisk (*) indicates $p<0.05$, and double asterisks (**) indicate $p<0.01$. ES was applied with 0.1 at 5 to 100 Hz for 1 h, and the CM was harvested 24 h later. (A&B) MTT-based viability of TT2 OS and PANC1 PDAC cells in response to ES-treated Jurkat CM.

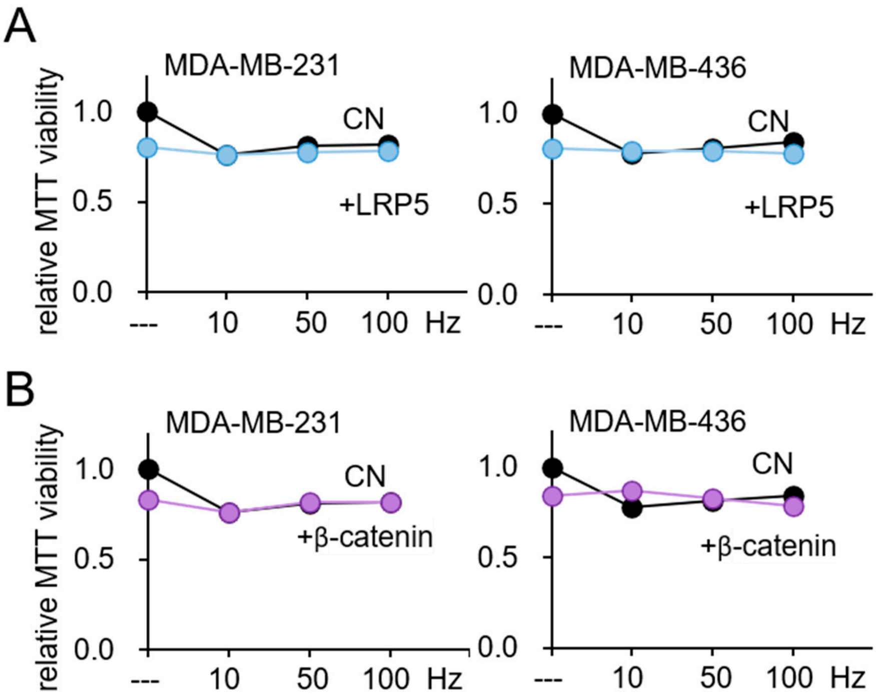

Figure S5. Unique Role of Wnt-signaling related proteins in ES. CN = control, and CM = conditioned medium. The single and double asterisks indicate $p < 0.05$ and 0.01, respectively. ES was applied with 0.1 V at 100 Hz for 1 h, and the CM was harvested 24 h later. (A) Reduction in MTT-based viability of MDA-MB-231 and MDA-MB-436 breast cancer cells in response to LRP5-overexpression MSC CM. But ES has no improvement in anti-cancer effect. (B) Reduction in MTT-based viability of MDA-MB-231 and MDA-MB-436 breast cancer cells in response to β-catenin-overexpression MSC CM. Similarly, ES has no improvement in the anti-cancer effect.

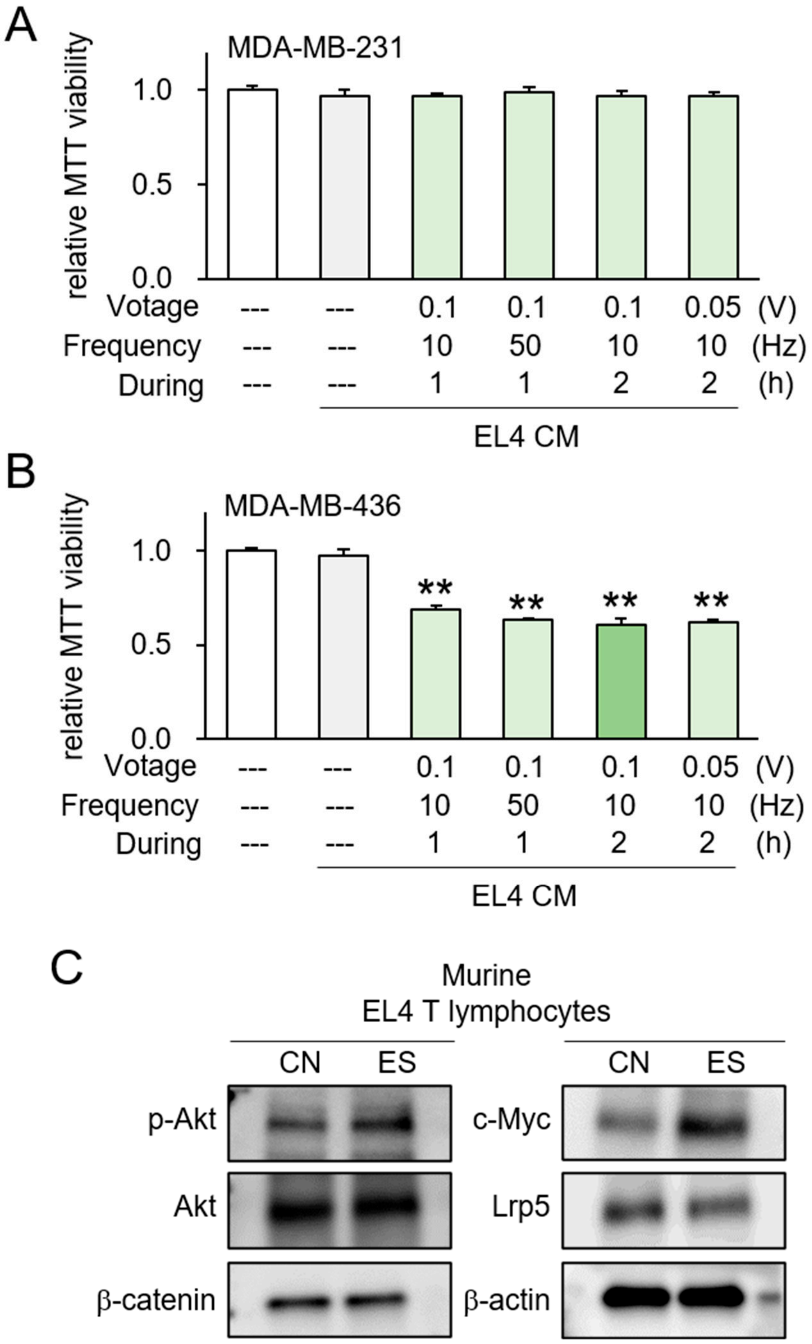

Figure S6. Tumor-suppressive effects of ES-treated EL4 CM. CN = control, and CM = conditioned medium. Data are presented as mean ± SD (n = 6 independent experiments). Statistical analysis was performed using one-way ANOVA followed by Bonferroni correction. The asterisks indicate statistical significance compared to the control group. Double asterisks (**) indicate $p<0.01$. ES was applied with 0.1 or 0.05 V at 10 or 50 Hz for 1 or 2 h, and the CM was harvested 24 h later. (A&B) MTT-based viability of MDA-MB-231 and MDA-MB-436 breast cancer cells in response to ES-treated EL4-derived CM. (C) Elevation of the levels of p-Akt, c-Myc in murine EL4 T lymphocytes in response to ES.

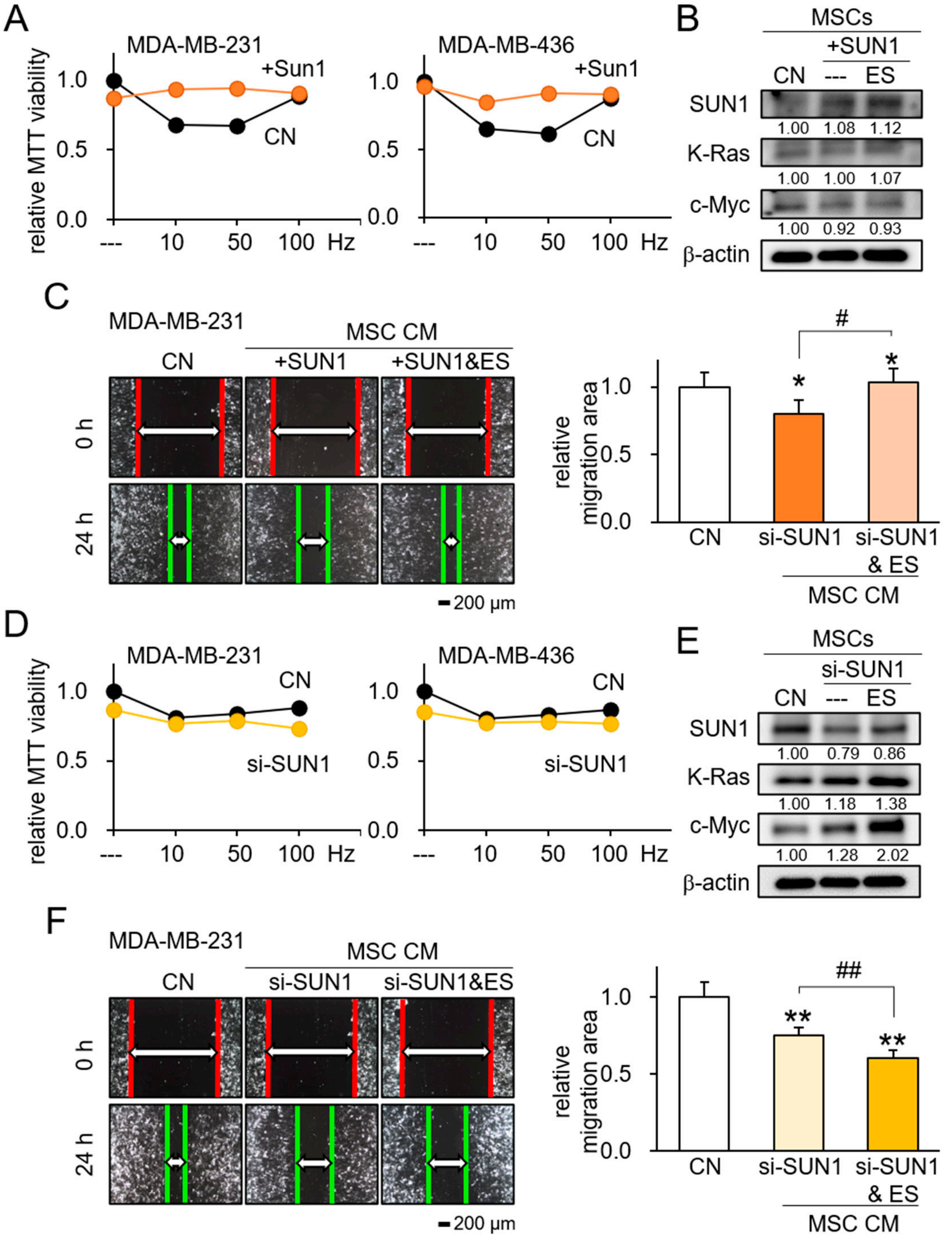

Figure S7. Unique Role of SUN1 in ES. CN = control, and CM = conditioned medium. Data are presented as mean ± SD (n = 6 independent experiments). Statistical analysis was performed using one-way ANOVA followed by Bonferroni correction. The asterisks indicate statistical significance compared to the control group. Single asterisk (*) indicates $p<0.05$, and double asterisks (**) indicate $p<0.01$. Using connecting lines to indicate group comparisons. Single pound sign (#) indicates $p < 0.05$, and double pound signs (##) indicate $p < 0.01$. ES was applied with 0.1 V at 100 Hz for 1 h, and the CM was harvested 24 h later. (A) MTT-based viability of

MDA-MB-231 and MDA-MB-436 breast cancer cells in response to ES-treated SUN1-overexpression MSC CM. (B) Elevated levels of SUN1 and decreased levels of β-catenin proteins in MSCs by the transfection of SUN1 plasmids. (C) Scratch-based motility of MDA-MB-231 cells in response to ES-treated SUN1-overexpression MSC CM. (D) MTT-based viability of MDA-MB-231 and MDA-MB-436 breast cancer cells in response to ES-treated si-SUN1-treated MSC CM. (E) Decreased levels of SUN1 and elevated levels of K-Ras and β-catenin proteins in MSCs by the transfection of si-SUN1 plasmids. (F) Suppression of scratch-based motility of MDA-MB-231 cells in response to ES-treated siSUN1 MSC CM.
